# Typhus in Old Age

**Published:** 1842-12-17

**Authors:** 


*Typhus in Old Age.*—Some years ago M. Chomel declared that he was not aware of a single authentic case of typhus fever in a person above the age of fifty-five. Since that period, M. Prus has reported an instance of a person seventy-four years of age who was affected with that disease; and M. Rayer has recently had a case in which the patient was fifty years of age when attacked.—*Lond. Lan.*

